# Distinctive expression of T cell guiding molecules in human autoimmune lymph node stromal cells upon TLR3 triggering

**DOI:** 10.1038/s41598-018-19951-5

**Published:** 2018-01-29

**Authors:** Janine S. Hähnlein, Tamara H. Ramwadhdoebe, Johanna F. Semmelink, Ivy Y. Choi, Ferco H. Berger, Mario Maas, Danielle M. Gerlag, Paul P. Tak, Teunis B. H. Geijtenbeek, Lisa G. M. van Baarsen

**Affiliations:** 1 Amsterdam Rheumatology & immunology Center (ARC), Clinical Immunology & Rheumatology, Academic Medical Center, University of Amsterdam, Amsterdam, The Netherlands; 2 Department of Experimental Immunology, Academic Medical Center, University of Amsterdam, Amsterdam, The Netherlands; 3 Department of Radiology, Academic Medical Center, University of Amsterdam, Amsterdam, The Netherlands; 4 Ghent University, Ghent, Belgium; 5 University of Cambridge, Cambridge, UK; 6 Present Address: Clinical Unit Cambridge, GlaxoSmithKline, Cambridge, UK; 7 GlaxoSmithKline, Stevenage, UK

## Abstract

Infections are implicated in autoimmunity. Autoantibodies are produced in lymphoid tissue where lymph node stromal cells (LNSCs) regulate lymphocyte function. Infections can alter the interaction between LNSCs and lymphocytes resulting in defective immune responses. In rheumatoid arthritis (RA) autoantibody production precedes clinical disease allowing identification of at risk individuals. We investigated the ability of human LNSCs derived from RA, RA-risk and healthy individuals to sense and respond to pathogens. Human LNSCs cultured directly from freshly collected lymph node biopsies expressed *TLR1-9* with exception of *TLR7*. In all donors TLR3 triggering induced expression of ISGs, IL-6 and adhesion molecules like VCAM-1 and ICAM-1. Strikingly, T cell guiding chemokines CCL19 and IL-8 as well as the antiviral gene *MxA* were less induced upon TLR3 triggering in autoimmune LNSCs. This observed decrease, found already in LNSCs of RA-risk individuals, may lead to incorrect positioning of lymphocytes and aberrant immune responses during viral infections.

## Introduction

Autoimmunity develops when tolerance mechanisms fail and self-reactive lymphocytes get activated in the periphery. Central tolerance occurs in the thymus and the bone marrow, but self-reactive lymphocytes can escape and should then be contained within secondary lymphoid organs. Through peripheral tolerance self-reactive lymphocytes become either functionally unresponsive (anergy), undergo clonal deletion after self-antigen presentation, become regulatory cells or are deprived of growth factors and stimulatory mediators^1,2^. In the lymph node (LN) these mechanisms of peripheral tolerance can be controlled by lymph node stromal cells (LNSCs)^3–10^.

Next to their crucial role in peripheral tolerance, LNSCs are also key players in modulating immune responses using other mechanisms. By the production of survival factors as IL-7 (interleukin 7) and BAFF (B cell activating factor; tumor necrosis factor superfamily member 13b or also known as B-lymphocyte stimulator (BLyS)), they regulate homeostasis of lymphocyte populations^11–13^. On the other hand, LNSCs can actively supress T cell proliferation by production of nitric oxide in an antigen-independent manner^14,15^. Through the production of extracellular matrix proteins LNSCs create the conduit system, a web of collagen-rich fibres that allows quick transport of soluble antigens and signaling molecules within the LN^16^. The conduits in turn are ensheathed by LNSCs thereby forming a dense network and creating the physical backbone of the LN. The expression of adhesion molecules and the production of fibres by LNSCs regulate entry and actively control the migration of B and T cells within the LN^17,18^. Together with the production of various chemokines such as CCL19, CCL21 and CXCL13 LNSCs attract, retain and position lymphocytes within the LN and thereby guide the interaction between T cells, B cells and antigen presenting cells^19–23^. Accordingly, it is essential that the function of LNSCs is tightly regulated and maintained during inflammation and infection.

In mice the exposure of LNSCs to microbial products changes their expression profile drastically^7,24^ and direct infection of LNSCs with LCMV (Lymphocytic Choriomeningitis Virus) leads to reduced activation of CD8 + T cells associated with delayed viral clearance^25^. Ablation of fibroblastic reticular cells (FRCs), one subset of LNSCs, deteriorates antiviral T cell responses, but also diminishes humoral immunity^13^. Furthermore, an effective antiviral response can only be mounted if LNSCs are able to expand and to mature during infection and are able to regenerate the LN structure after resolving acute inflammation^26–28^. Chronic infection and inflammation leads to fibrosis of the LN which interferes with these processes and can consequently contribute to further deregulation of the immune system^29^. Given the importance of LNSCs in modulating immune responses we hypothesize that malfunctioning of LNSCs may lead to a LN microenvironment where immune responses are not properly controlled possibly resulting in autoimmune disorders.

Our knowledge on LNSCs is mainly based on mouse studies, human studies are lacking. Here we developed an experimental model to investigate the response of human LNSCs to viral infection during very early stages of systemic autoimmunity. We use rheumatoid arthritis (RA) as a model autoimmune disease since the production of the RA-specific autoantibodies IgM rheumatoid factor (IgM-RF) and anti-citrullinated protein antibodies (ACPAs) can be detected years before clinical onset of disease^30^. This allows for the selection of individuals at risk for developing RA (RA-risk individuals) and thus to study the earliest phases of systemic autoimmunity without the interference of an inflammatory clinical disease^31^. In the peripheral blood of a subset of RA patients an increased expression of type I interferon (IFN) response genes (IGSs) has been reported^32,33^ and this signature is also present in at risk individuals who developed RA after follow up^34,35^. Moreover, disturbances in Toll-like receptor (TLR)^36,37^ signaling as well as the presence of multiple viral species in the synovial compartment of early RA patients^38^ suggest a role for viral responses in this autoimmune disease. Here we show that the TLR3 induced production of T cell guiding chemokines by human LNSCs is diminished at a very early stage of systemic autoimmunity.

## Results

### Functional responsiveness of human LNSCs to TLR3 triggering

**Figure 1:**
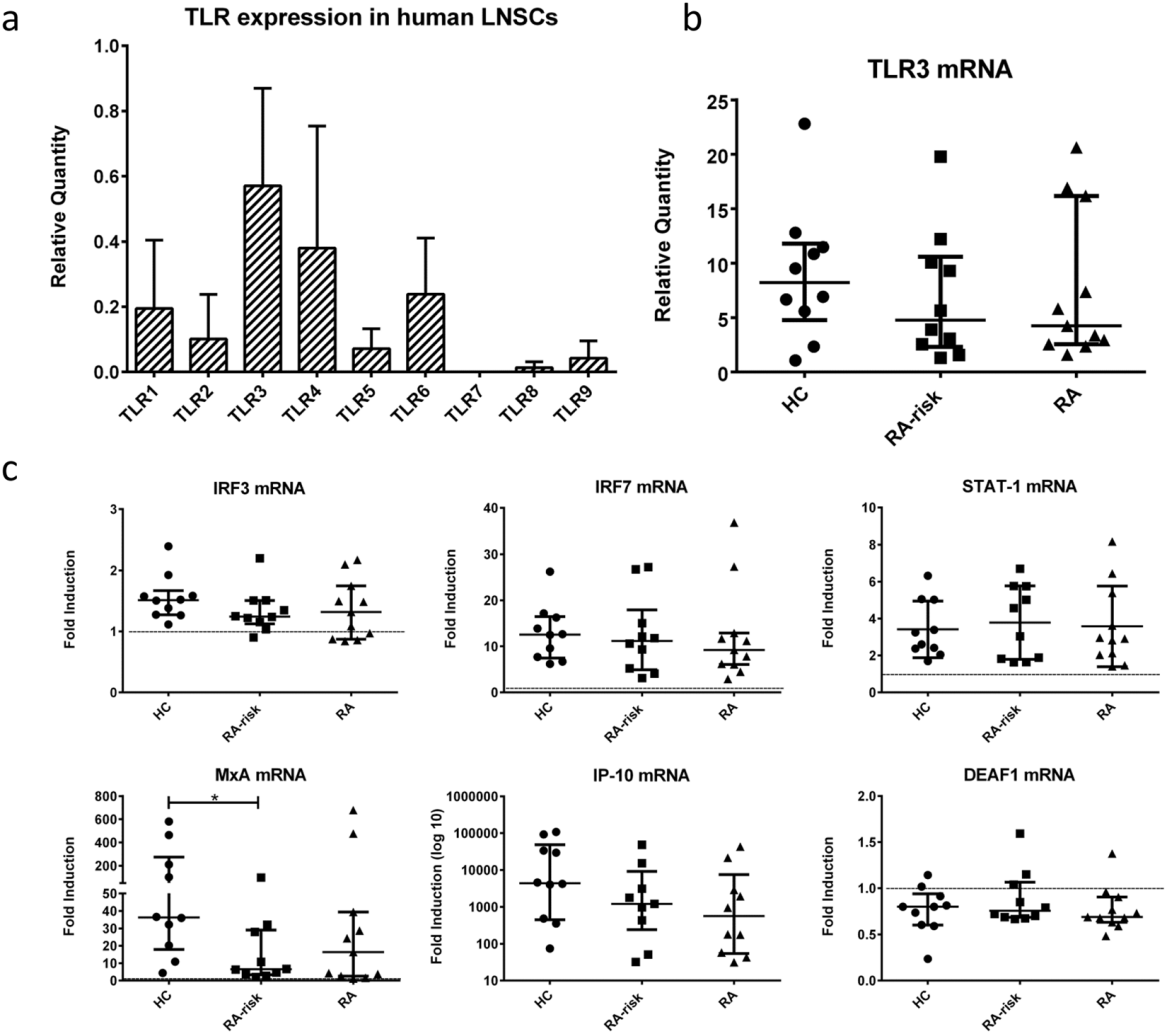
TLR expression in human LNSCs and induction of IFN-stimulated genes (ISGs). (**a**) mRNA expression levels of different TLRs were assessed in a small cohort by qPCR and data are presented as mean plus standard deviation in five donors (healthy n = 3 and RA n = 2). (**b**) TLR3 expression in LNSCs of 31 donors was measured by qPCR (healthy n = 10, RA-risk n = 10 and RA n = 11). Data are presented as median plus interquartile range. Expression levels were normalized to a different calibrator sample resulting in different relative quantities compared to Fig. 1a. (**c**) The induction of ISGs in human LNSCs after stimulation with poly(I:C) for 48 hours was assessed by qPCR. The mRNA levels of *IRF3* (IFN response factor 3), *IRF7* (IFN response factor 7), *IFNB1* (Interferon beta 1), *STAT1* (signal transducer and activator of transcription 1), *MxA* (myxoma resistence protein), *IP-10* (IFNγ-inducible protein 10, also known as CXCL10) and *DEAF1* (deformed epidermal autoregulatory factor 1) were measured. Data are represented as the fold induction (median with interquartile range) by comparing the expression in stimulated cells to corresponding unstimulated cells in 31 donors (healthy n = 10, RA-risk n = 10 and RA n = 11). Differences between donor groups were assessed by Kruskal-Wallis followed by a post Dunn’s test. *P < 0.050. The dotted line represents a fold induction of 1.

We first investigated the expression of TLRs in human LNSCs to assess their capacity to sense pathogens. Under homeostatic conditions we detected mRNA for *TLR1-9* in a small cohort of 5 donors with the exception of *TLR7*. *TLR3* showed the highest and most robust expression levels which on average were not different between healthy, RA-risk and RA donors (Fig. 1a,b). We therefore chose to trigger TLR3 with poly(I:C), which can be equally sensed by all donors, to simulate a viral infection in LNSCs after which we compared the response between healthy, RA-risk and RA donors.

We measured the induction of genes downstream of TLR3 signaling to evaluate the functional responsiveness of TLR3 in cultured LNSCs from healthy, RA-risk and RA donors. We tested the induction of *IRF3* (IFN response factor 3) which is a required IFN response element for TLR3 signaling, and ISGs *IRF7* (IFN response factor 7), *IFNB1* (Interferon beta 1), *STAT1* (signal transducer and activator of transcription 1), *MxA* (myxoma resistence protein), *IP-10* (IFNγ-inducible protein 10, also known as CXCL10) and *DEAF1* (deformed epidermal autoregulatory factor 1) which has recently been identified to participate the TLR3 signaling pathway^39^ (Fig. 1c). Under homeostatic conditions no differences in expression levels were observed between donor groups. Most genes tested were upregulated in response to poly(I:C) with exception of *DEAF1* which was slightly decreased in all donor groups after stimulation. *IRF3* was clearly detected in all LNSC donors and the expression did not change upon TLR3 triggering. Using this experimental set-up we could not detect *IFNB1* mRNA (data not shown). However, *IRF7*, *STAT1*, *MxA* and *IP-10* were highly induced upon TLR3 stimulation. Of note, for the antiviral gene *MxA* we detected a significantly lower induction (P = 0.0444) in RA-risk individuals compared with healthy controls.

**Figure 2:**
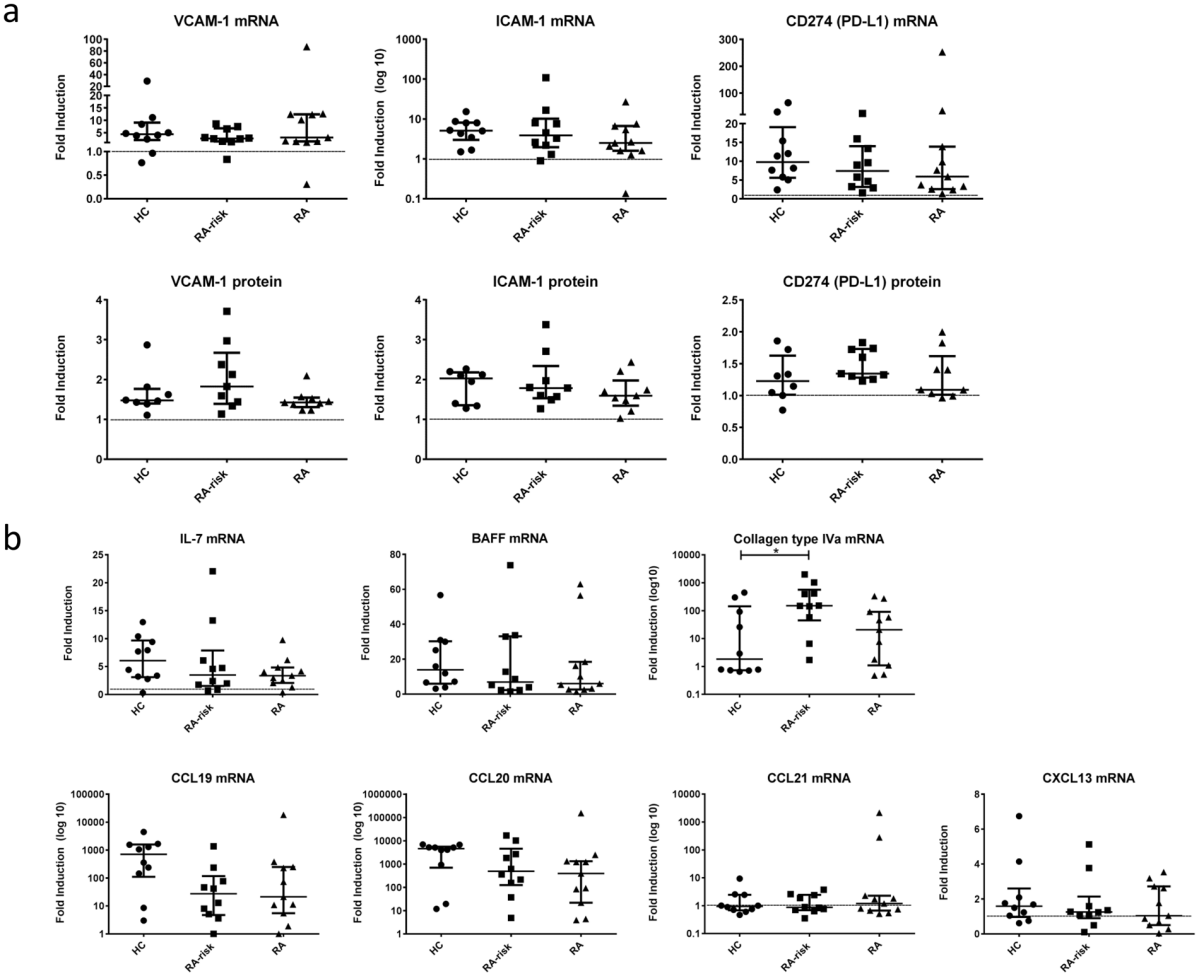
Poly(I:C) mediated induction of genes involved in cell-cell contact and lymphocyte positioning. (**a**) Induction of surface molecules VCAM-1, ICAM-1 and PD-L1 was assessed after stimulation with poly(I:C) using qPCR (48 h) and FACS (24 h) analysis. Data are represented as the fold induction (median with interquartile range) by comparing the expression in stimulated cells to corresponding unstimulated cells in 31 donors (healthy n = 10, RA-risk n = 10 and RA n = 11) for qPCR analysis and in 26 donors (healthy n = 8, RA-risk n = 9 and RA n = 9) for FACS analysis. (**b**) Induction of *IL-7*, *BAFF*, *collagen type IVa, CCL19, CCL20, CCL21* and *CXCL13* was measured by qPCR after 48 h stimulation with poly(I:C). Data are represented as the fold induction (median with interquartile range) by comparing the expression in stimulated cells to corresponding unstimulated cells in 31 donors (healthy n = 10, RA-risk n = 10 and RA n = 11). Differences between donor groups were assessed by Kruskal-Wallis followed by a post Dunn’s test. *P < 0.050. The dotted line represents a fold induction of 1.

Subsequently, we analyzed the induction of molecules involved in direct T cell contact, like VCAM-1 (vascular cell adhesion molecule 1) and ICAM-1 (intercellular adhesion molecule 1) and the negative T cell regulator PD-L1 (CD274; programmed cell death ligand 1). In response to TLR3 triggering the expression of VCAM-1, ICAM-1 and PD-L1 was in most donors clearly upregulated at mRNA as well as protein level (Fig. 2a). No differences were detected between the donor groups, neither in fold induction nor under homeostatic conditions.

### Autoimmune LNSCs produced less T cell guiding chemokines upon TLR3 triggering

**Figure 3:**
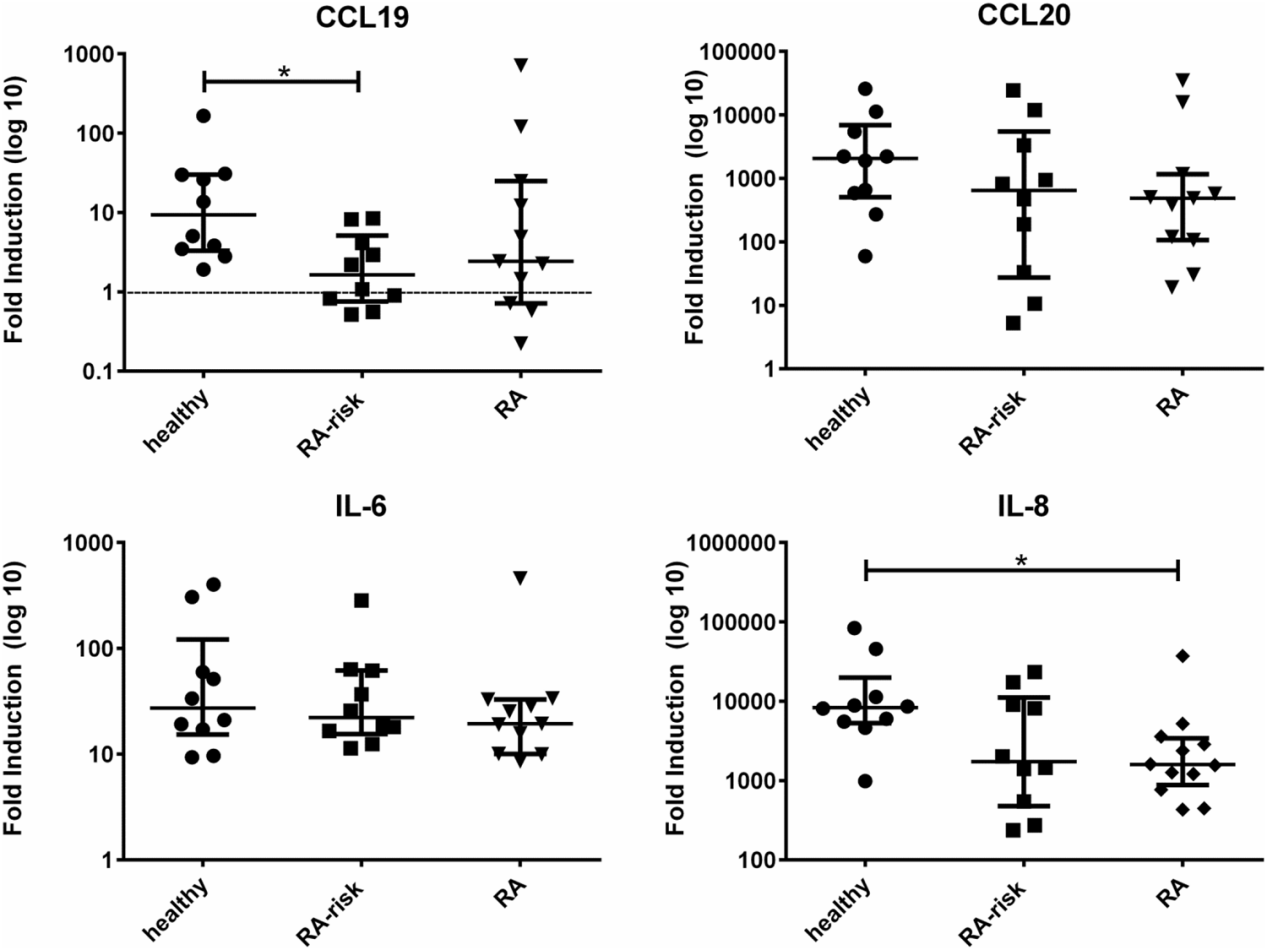
Induction of T cell positioning chemokines and pro-inflammatory cytokines measured on protein level. Levels of CCL19, CCL20, IL-6 and IL-8 in supernatants of LNSCs were assessed on protein level by ELISA after 48 h stimulation with poly(I:C). Concentrations measured for CCL19 ranged between 2–700 pg/ml, for CCL20 between 0.002–60 ng/ml, for IL-6 between 0.6–400 ng/ml and for IL-8 between 0.005–600 ng/ml. Data are represented as fold induction (median with interquartile range) by comparing the protein levels in stimulated cells to corresponding unstimulated cells in 31 donors (healthy n = 10, RA-risk n = 10 and RA n = 11). Differences between donor groups were assessed by Kruskal-Wallis followed by a post Dunn’s test. *P < 0.050. The dotted line represents a fold induction of 1.

Next we investigated the response to TLR3 triggering for molecules characteristic of LNSCs (Fig. 2b). LNSCs are a prominent source of IL-7 and BAFF in the LN ensuring T cell and innate lymphoid cell (ILC)^28,40^ as well as B cell survival^13,41^. For the formation of the conduit network, which provides physical guidance, LNSCs produce a great variety of extracellular matrix molecules such as collagen type IVa^24^. We furthermore included chemokines responsible for attraction and positioning of lymphocytes and dendritic cells in the LN microenvironment such as CCL19 (C-C motif chemokine ligand 19), CCL20 (C-C motif chemokine ligand 20), CCL21 (C-C motif chemokine ligand 21) and CXCL13 (C-X-C motif chemokine ligand 13)^22^. For *IL-7* and *BAFF* we detected a clear upregulation after poly(I:C) stimulation, with no differences between donor groups. For *COL4A1* mRNA we observed a significantly higher induction in RA-risk individuals compared with healthy controls (P = 0.0389). All chemokines measured were detected at very low levels in LNSCs under homeostatic conditions but all, except for *CCL21*, were clearly induced upon TLR3 stimulation. Overall the response to TLR3 triggering was very variable between individuals, however we observed a non-significant lower induction in individuals with systemic autoimmunity in comparison with healthy controls for *IL-7*, *CCL19, CCL20* and to a lesser extent for *CXCL13*. This trend was more pronounced for *CCL19* and *CCL20*. To verify these findings on the protein level we performed ELISAs for CCL19 and CCL20 (Fig. 3). Basal protein levels for these two chemokines were undetected or very low, but clearly induced upon TLR3 triggering. Concentrations measured for CCL19 ranged between 2–700 pg/ml and for CCL20 between 0.002–60 ng/ml. We found significantly lower induction of CCL19 protein secretion by LNSCs obtained from RA-risk individuals compared with healthy controls (P = 0.0298). In addition to chemokines, we also measured the production of pro-inflammatory cytokines IL-6 and IL-8 on protein level and we found a significantly lower induction of IL-8 in LNSCs of RA patients compared with healthy controls (P = 0.025) (Fig. 3). IL-6 induction was similar in all donor groups. No differences were found under homeostatic conditions. For IL-6 we measured concentrations between 0.6–400 ng/ml and for IL-8 between 0.005–600 ng/ml.

Finally, we investigated whether the observed findings were related to clinical parameters like autoantibody production or age. ACPA status, age and other disease parameters did not correlate with the expression or induction of genes and proteins investigated in this study with exception of CCL19. The lower induction for CCL19 protein in RA-risk individuals was stronger in ACPA positive individuals (P = 0.0304 for mRNA levels and P = 0.0098 for protein levels), but remained non-significant in ACPA positive RA patients (Supplementary Figure S1).

## Discussion

Since our knowledge of the role of LNSCs in the immune response is mainly based on mouse studies we developed an experimental model to investigate the response of human LNSCs to TLR triggering during very early stages of systemic autoimmunity. The results presented here show that human LNSCs express various TLRs and are responsive to TLR3 triggering using poly(I:C) treatment. Strikingly, upon TLR3 stimulation, human LNSCs derived from RA-risk individuals or RA patients exhibit a lower induction of some antiviral genes, chemokines and cytokines compared with healthy controls, thus pointing towards a deregulated antiviral response of LNSCs during systemic autoimmunity already present in patients at risk for RA.

We observed a significantly lower induction of the antiviral gene *MxA* in LNSCs from RA-risk individuals. MxA, also called MX Dynamin Like GTPase 1, is a key effector protein dependent solely on IFN signaling and inhibits replication of a broad range of viruses^42^. For example, human lung carcinoma cells stimulated with IFN-α show abolished suppression of influenza A virus replication after knocking down of MxA by siRNA^43^. Decreased induction of MxA might therefore lead to increased viral replication in LNSCs, which can change their function in various ways while exploiting LNSCs as possible virus reservoir. So far the direct infection of LNSCs has been investigated solely in mice and nonhuman primates where haemorrhagic fever viruses as Ebola, Marburg and Lassa target FRCs^44^. Although RA is not associated with these haemorrhagic fever viruses, several other viruses have been proposed to be involved in RA development^45^. The most prominent candidate is Epstein-Barr virus (EBV) for which increased titres are found in RA patients compared with healthy controls^46,47^. Therefore the precise underlying mechanism and possible defects in viral clearance in autoimmune LNSCs as well as their potential to contribute to an impaired host defence and the increased infection risk seen in RA patients^48^ remains to be determined.

Moreover, it is tempting to speculate that LNSCs in the very earliest phases of autoimmunity display a protective behaviour after poly(I:C) treatment, possibly as an attempt to inhibit further activation of the immune system. In autoimmune LNSCs we observed a lower induction of lymphocyte guiding chemokines, most robustly seen for CCL19 and IL-8, but with a similar response pattern for IL-7, CCL20 and CXCL13. The chemokines CCL19 together with CCL21 (both ligands for CCR7) and CXCL13 (ligand for CXCR5) dictate the homing and orchestrate positioning of lymphocytes into the T cell area or B cell follicles of the LN^22,49,50^. Naïve T cells highly express CCR7 and its signaling enhances synapse formation with DCs and hence helps in T cell activation^51^. CXCR5 is highly expressed on mature B cells, which upon antigen binding induces the expression of CCR7. On the other hand, follicular T helper cells downregulate CCR7 and upregulate CXCR5. Hence, both cell types migrate to the follicular border to initiate germinal center (GC) responses following the chemokine gradient^52,53^. In addition, IL-8 might also be involved in recruitment of CXCR1 positive T cells into GC^54,55^ and it has been shown to pronounce survival of malignant B cells^56^. IL-8 is known primarily as attractant of neutrophils, which are rapidly recruited to the LN after infection and can influence T cell proliferation and polarization^57^. However, studies also showed that CXCR2, an IL-8R expressed on neutrophils, plays a minor role in neutrophil recruitment to the LN^58^ and their exact role in the LN is unclear. Also CCL20 and CXCL13, chemokines attracting mainly CCR6+ memory T cells and B cells respectively^59,60^, are less induced by autoimmune LNSCs upon TLR-3 stimulation. This lower induction might disturb the chemokine gradient within the LN to such an extent that lymphocytes might be misplaced. Moreover lower chemokine expression in LNSCs might lead to decreased activation of lymphocytes and deregulated GC responses because B and T helper cells are not correctly guided towards their interaction partners at the follicular border and lymphocytes may have a disturbed retention time within the LN. Indeed in mouse lymphoid tissue specifically the homeostatic chemokines CCL19, CCL21 and CXCL13 were downregulated after infection with several different pathogens leading to altered homing, positioning and priming of lymphocytes within the LN^61^. The authors suggest that by limiting the amount of incoming lymphocytes, cellular integrity of the LN is protected and might be necessary for terminating the ongoing immune response^61^. Moreover, another study in mice, in which FRCs were conditionally depleted, showed that attraction and retention of naïve CD8+ T cells was dependent on CCL19 and CCL21 expressing FRCs, while retention of activated CD8+ T cell was regulated otherwise^62^. Interestingly, our results are in line with our previous findings on changes in frequency and activation state of lymphocyte populations within LNs of RA-risk and RA patients when compared with healthy controls^63–65^. RA(-risk) patients have a decreased frequency of lymph node CD4+ CD45RA+ naïve T cells as well as a lower frequency of CCR7 expressing CD4+ and CD8+ T cells in comparison to healthy controls^63,64^, which is in line with the decreased capacity to produce CCL19 by LNSCs during the earliest phases of RA. During systemic autoimmunity, the lower production of lymphocyte guiding chemokines by human LNSCs upon TLR stimulation might be an attempt to prevent influx and activation of autoreactive lymphocytes, although this warrants further investigation.

The underlying mechanism of this observed reduced TLR3 response in LNSCs of RA-risk and RA patients remains to be identified. The reason might be an intrinsic, unknown error in autoimmune LNSCs or we might hypothesize that LNSCs, similarly as T cells, become exhausted during systemic autoimmunity^66^. In addition, fibroblast-like synoviocytes (FLS) responses are known to depend on positional^67^ and inflammatory memory^68^, a phenomenon which might also control LNSC responses. Interestingly, osteoclasts can be directly activated by monoclonal ACPAs leading to increased bone absorption and IL-8 production^69^. It will be of interest to investigate whether ACPAs can also activate LNSCs resulting in an exhausted phenotype already during the RA-risk phase of disease. These potential mechanisms will be subject of future research.

A further observation supporting a protective role of LNSCs in autoimmunity after TLR3 triggering is provided by the significant higher induction of *COL4A1* (collagen type IV alpha 1 chain) in RA-risk individuals compared with healthy controls and the trend towards lower induction of *IL-7* in individuals with systemic autoimmunity. IL-7 is a crucial cytokine in adaptive immunity, it plays among others a pivotal role in remodelling of the LN after infection^28^, in induction of T follicular helper cells and humoral immunity^70^, but IL-7 is mainly known as is a survival factor for naïve and memory T cells^71^. During chronic inflammation regulatory T cells are known to produce TGF-β1 (transforming growth factor-β1) which in turn stimulates LNSCs to increase collagen synthesis. Through excessive collagen production by LNSCs T cells loose contact with the LNSCs and IL-7 gets captured and inaccessible causing T cell apoptosis^29^. Accordingly, the increased collagen type IVa and decreased IL-7 production by TLR3 stimulated LNSCs might be an attempt of activated LNSCs to inhibit (autoreactive) T cells during the RA-risk phase and it will be of interest to investigate this in future studies.

In summary, we show for the first time that, upon TLR3 triggering, human LNSCs from RA-risk individuals and RA patients produce less T cell guiding chemokines as well as the antiviral molecule MxA compared with healthy controls. This data may suggest that during systemic autoimmunity human LNSCs attempt to inhibit immune responses when activated through TLR3 and possibly other receptors. Since it is difficult to obtain LN biopsies from a large cohort of RA-risk individuals and RA patients and the culture of human LNSCs is very time consuming because of their slow growth, the number of donors analysed in this study is relatively low. Another important point is the high variability between donors which may originate from the heterogeneous LNSC population. These data warrant further validation studies to further address how we can exploit the mechanism by which LNSCs dampen immune responses and induce tolerance.

## Methods

### Study subjects and lymph node biopsy sampling

We included 17 individuals who had arthralgia and elevated IgM-RF and/or ACPA serum levels, without any evidence of arthritis upon clinical examination (RA-risk individuals, phase c/d^31^). IgM-RF was measured using IgM-RF ELISA (Hycor Biomedical, Indianapolis, IN (ULN 49 IU/mL)). ACPA was measured using anti-CCP2 ELISA CCPlus (Eurodiagnostica, Nijmegen, the Netherlands (ULN 25 kAU/L)). After a median follow up time of 25.6 months (13.6–38.7 (IQR)) none of these RA-risk individuals had developed RA yet despite the presence of autoantibodies. However, we expect that approximately 50% of these individuals will develop arthritis within 3–4 years^72,73^. These individuals are termed RA-risk individuals as recommended by the Study Group for Risk Factors for RA (SGRFRA) under the auspices of the EULAR (the European League Against Rheumatism) Standing Committee of Investigative Research (ESCIR)^31^. RA-risk individuals were not allowed to have systemic or intra-articular corticosteroid injection less than 28 days before enrolment. Furthermore, we included 17 RA patients with established disease based on fulfillment of the American College of Rheumatology and European League Against Rheumatism (ACR/EULAR) 2010 criteria^74^ as well as 10 healthy controls without any joint complaints and without elevated IgM-RF and/or ACPA levels. These healthy controls did not have a recent history of viral infection, no autoimmunity or malignancy and no present or previous use of DMARDs (disease-modifying antirheumatic drugs), biologicals or experimental drugs. The study was performed according to the principles of the Declaration of Helsinki, approved by the institutional medical ethical review board of the Academic Medical Centre, and all study subjects gave written informed consent. All study subjects underwent an ultrasound-guided inguinal LN needle core biopsy as previously described^75^. At the day of LN sampling none of the donors showed signs of an infection. Table 1 shows the demographics of the included subjects.

**Table 1:** Demographic data of study subjects.

	Healthy controls n = 10	RA-risk individuals n = 17	RA patients n = 17
Sex (female) (n) (%)	7 (70)*	15 (88)	11 (65)
Age (years) (median (IQR)	31 (27–39)	50 (37–56)	57 (44–61)
IgM-RF positive (n) (%)	0 (0)	6 (35)	14 (82)
IgM-RF level (kU/l) (median (IQR)	—	16 (5–87)	109 (17–280)
ACPA positive (n) (%)	0 (0)	11 (75)	14 (82)
ACPA level (kAU/l) (median (IQR)	—	60 (4–190)	274 (48–1331)
IgM-RF and ACPA both positive (n) (%)	0 (0)	0 (0)	11 (65)
ESR (mm/h) (median (IQR))	nd	5 (2–10)	9 (4–25)^b^
CRP (mg/l) (median (IQR))	0.5 (0.4–2.2)^a^*	1.9 (0.6–4.25)	6.8 (1.53–17)^c^
68 TJC (n) (median (IQR)	0 (0)	1 (0–5)	7.5 (3.5–17)^d^
66 SJC (n) (median (IQR))	0 (0)	0 (0)	3 (0–10)^d^
DAS28 (median (IQR))	—	—	4.2 (3.1–5.1)^a^
Medication (n) (%)	—	—	8 (47)
• Corticoid	—	—	5 (29)
• NSAID	—	—	4 (24)^f^
• DMARD	—	—	6 (35)
• Failed TNF therapy	—	—	3 (18)

Categorical variables: n (%) Continuous variables (data not normally distributed): median (IQR). *Healthy controls are significantly younger and have lower CRP levels than RA patients (P < 0.050, tested by Kruskal-Wallis followed by a post Dunn’s test). ^a^levels missing from 1 individual, ^b^levels missing from 4 individuals, ^c^levels missing from 5 individuals, ^d^levels missing from 3 individuals, ^f^levels missing from 4 individuals.

IgM-RF, rheumatoid factor; ACPA, anti-citrullinated protein antibodies ESR, erythrocyte sedimentation rate; CRP, C-reactive protein; 68 TJC, tender joint count of 68 joints; 66 SJC, swollen joint count of 66 joints; nd, not determined.

### Lymph node stromal cell culture and stimulation

After depletion of lymphocytes through a 70 μm cell strainer (BD Falcon, San Jose, CA) the remaining stromal tissue of a freshly collected LN needle core biopsy was plated on a 6-well culture dish (Greiner CELLSTAR®, Sigma Aldrich, Zwijndrecht, the Netherlands) (passage 0; P0). Complete cell culture medium was added which consists of Dulbecco’s Modified Eagle Medium (DMEM) low glucose (Gibco, Bleiswijk, the Netherlands) supplemented with 0.1% penicillin (Astellas Pharma Inc, Leiden, the Netherlands), 0.1% streptomycin, 0.05 mg/mL gentamicin, 10 mM HEPES buffer, 2 mM L-glutamine (all Gibco) and 10% fetal calf serum (FCS) (GE Healthcare, Zeist, the Netherlands). Human LNSCs formed monolayers and for expansion were passaged using trypsinisation (0.05% trypsin, 5 mM EDTA (Gibco)) in phosphate buffer saline (PBS, Fresenius Kabi,‘s Hertogenbosch, the Netherlands) for 7 minutes at 37 °C. For harvesting, cells were washed with warm sterile PBS, trypsinized and the cell suspension was collected and centrifuged for 10 minutes, 1000 rpm at 4 °C. Cells were resuspended in cold complete medium and counted using trypan blue (Sigma Aldrich) in a Burker Turk chamber (LO Labor Optik, Lancing, UK). Subsequently, human LNSCs were seeded in a 6-well plate (100,000–200,000/well) and stimulated with polyinosinic–polycytidylic acid (poly(I:C)) (20 ug/ml, Sigma Aldrich). Simultaneously we phenotyped LNSCs by flow cytometry based on expression of CD45, Podoplanin (gp38) and CD31. LNSCs in culture were double negative cells (DNs; PDPN-CD31-) and fibroblastic reticular cells (FRCs; PDPN+ CD31−) (Supporting Information Figure S2).

### Flow cytometry analysis

Human LNSCs (passages 3 to 6) were harvested from a 6-well dish using one ml TripLE™ Select (Gibco) for 10 minutes at 37 °C. Subsequently, cells were washed in PBA buffer (PBS containing 0.01% NaN_3_ and 0.5% bovine serum albumin (BSA) (Sigma Aldrich)) and stained for 30 minutes at room temperature protected from light using the following directly labelled antibodies: ICAM-1 (CD54) PerCP (clone 1H4, EXBIO, Huissen, the Netherlands), VCAM-1 (CD106) PE (clone STA, eBioscience, Vienna, Austria), CD254 (PD-L1) FITC (clone MOPC-21, Becton Dickinson (BD) Pharmingen, Breda, the Netherlands) with corresponding isotypes. Staining with HLA-ABC Pe-Cy7 (clone G46-2.6, Biolegend, London, UK) served as a positive control. For phenotyping LNSCs in culture we used the following directly labelled antibodies: CD45 FITC (clone HI30, BD Pharmingen), Podoplanin AlexaFluor647 (clone NC-08, Biolegend), CD31 APC-eFluor780 (clone WM-59, eBioscience) (Supplementary Figure S2). Then cells were washed in PBA and measured on a FACS CANTO II (BD). Data were analysed using FlowJo software 9.9.3 (Tree Star, Ashland, OR). Fold induction was calculated using the mean fluorescence intensity (MFI) by first taking the ratio between staining and isotype control for both conditions and then calculating the ratio between stimulated and unstimulated condition according to the following formula: [MFI stimulated stained/MFI stimulated isotype control]/[MFI unstimulated stained/MFI unstimulated isotype control].

### Quantitative real-time PCR

mRNA was isolated using the RNeasy Mini kit or RNeasy Micro kit (Qiagen, Venlo, the Netherlands) according to the manufacturer’s instructions, including a DNAse step to remove genomic DNA. Subsequently cDNA was prepared using the RevertAid H Minus First Strand cDNA Synthesis kit (Thermo Fisher Scientific, Landsmeer, the Netherlands). Quantitative PCR was performed using either Taqman® Universal PCR master mix combined with Taqman assays or SYBR® Green PCR master mix (all from Applied Biosystems, Life Technologies, Zwijndrecht, the Netherlands) combined with in house designed primers (Thermo Fisher Scientific). Taqman assays and primer sequences are described in Supplementary Table S3. For detection we used a StepOnePlus™ Real-Time PCR System (Applied Biosystems). Values for each target gene were corrected by the expression level of 18 S RNA. An arbitrary calibrator sample was used for normalization. For calculating the relative quantity (RQ) the delta-delta Ct method was used for Taqman assays and a standard curve method was applied for SYBR green assays. The fold induction was calculated using the following formula: [RQ stimulated/RQ unstimulated].

### Chemokine analysis by enzyme-linked immunosorbent assay (ELISA)

Supernatants of human LNSCs were harvested after 48 hours of stimulation with poly(I:C) (20 ug/ml). ELISAs were performed in clear flat-bottom 96-well Maxisorp Nunc-Immuno plates (Thermo Fisher Scientific) according to the manufacturer’s instructions. Reagents of ELISA for human IL-6 and IL-8 were purchased from Sanquin (Pelikine compact kit, Amsterdam, the Nederlands) and reagents for CCL19 and CCL20 were bought from R&D Systems (Duoset ELISA, Abingdon, UK). Absorbance was measured using the dual filter 450/540 substract settings on the microplate reader model 680 and data was displayed in the microplate manager 5.2 program, both from Bio-Rad Laboratories (Hercules, California, USA). Each donor and condition was measured in triplicate.

### Statistics

Data are presented as median with interquartile range or mean with standard deviation, where appropriate. Differences between study groups were analysed using Kruskal-Wallis test followed by a Dunn’s multiple comparison test. GraphPad Prism software (V.6, La Jolla, CA) was used for statistical analysis. P-values < 0.05 were considered statistically significant.

### Data availability statement

All data generated or analysed during this study are included in this published article (and its Supplementary Information files).

### Ethics approval and formal consent

The medical ethical committee of the Academic Medical Center Amsterdam approved this study and all study subjects gave written informed consent.

## Electronic supplementary material

Supplementary Information
