# Supplementary material for: Distinctive expression of T cell guiding molecules in human autoimmune lymph node stromal cells upon TLR3 triggering
Source: Sci Rep. 2018 Jan 29;8:1736. doi: 10.1038/s41598-018-19951-5 (PMC5789053; doi:10.1038/s41598-018-19951-5)
Supplement: Supplementary file 1 — Supplementary Information [file 41598_2018_19951_MOESM1_ESM.doc]

**Supplementary Information**

**Distinctive expression of T cell guiding molecules in human autoimmune lymph node stromal cells upon TLR3 triggering**

Co-authors:
Janine S Hähnlein^1,2^, Tamara H Ramwadhdoebe ^1,2^, Johanna F Semmelink^1,2^, Ivy Y Choi^1^, Ferco H Berger^3^, Mario Maas^3^, Danielle M Gerlag^1,4^, Paul P Tak^1,5^ , Teunis B H Geijtenbeek^2^, Lisa G M van Baarsen1,2*

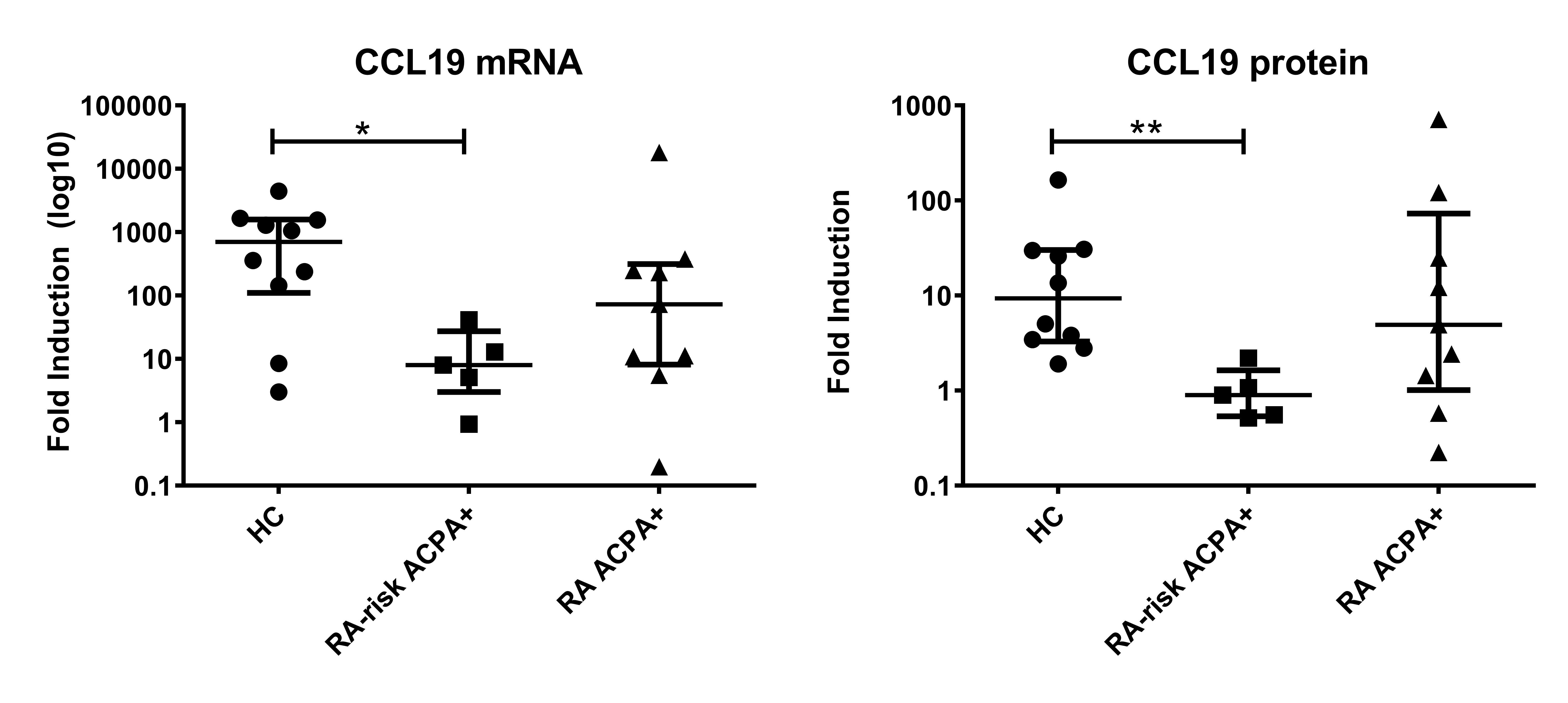


**Supplementary Figure S1**: CCL19 mRNA and protein expression in healthy controls compared with ACPA positive individuals
Levels of CCL19 mRNA were assessed by qPCR and protein was measured in supernatants of LNSC by ELISA after 48h stimulation with poly(I:C). Data are represented as the fold induction (median with interquartile range) by comparing stimulated cells to corresponding unstimulated cells in 24 donors (healthy n=10, RA-risk n=5 and RA n=9). Differences between donor groups were assessed by Kruskal-Wallis followed by a post Dunn’s test. * p<0.050 ** p< 0.010 **
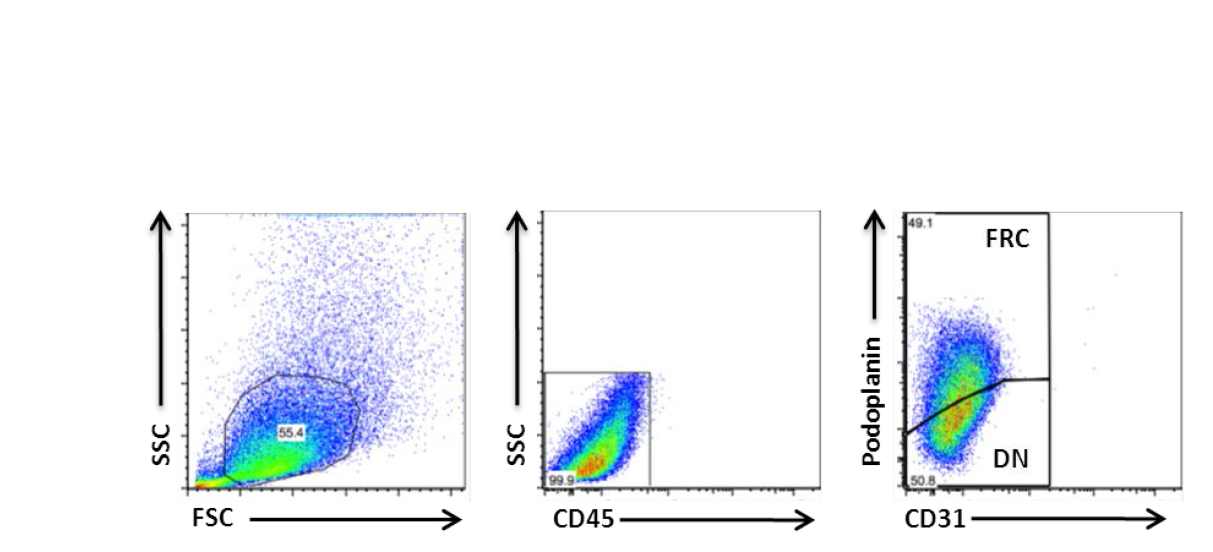
**

**Supplementary Figure S2**: Phenotypic characterization of cultured human LNSCs
Flow cytometry analysis using multi-colour approach, gating on CD45, Podoplanin (gp38) and CD31. Stromal cells in culture are DN cells (PDPN-CD31-) and FRCs (PDPN+CD31-). Gating was based on isotype controls. Representative figures of one donor out of 26 experiments are shown.

**Supplementary Table S3: Primers used in this study.**

| **Gene** | **Taqman ID** |  |
| --- | --- | --- |
| Deaf1 | Hs00221402_m1 |  |
| ICAM-1 | Hs00164932_m1 |  |
| CD274 (PD-L1) | Hs01125301_m1 |  |
| IL-7 | Hs00174202_m1 |  |
| CCL19 | Hs00171149_m1 |  |
| CCL20 | Hs01011368_m1 |  |
| CCL21 | Hs00989654_g1 |  |
| CXCL13 | Hs00757930_m1 |  |
| 18S RNA | Hs99999901_s1 |  |
| **Gene** | **Forward Primer sequence 5’-3’** | **Reverse Primer sequence 5’-3’** |
| TLR1 | TGCTGCCAATTGCTCATTTG | GAAGGCCCTCAGGGTCTTCT |
| TLR2 | GGCTTCTCTGTCTTGTGACCG | GAGCCCTGAGGGAATGGAG |
| TLR3 | TGTTCAGAAAGAGGCCAAATAATCT | CATCGAATCAAATTAAAGAGTTTTCTCCAGGGTG |
| TLR4 | CTGCAATGGATCAAGGACCAG | CCATTCGTTCAACTTCCACCA |
| TLR5 | GCTGGTGCCTTGAAGCCTT | GACCCAACCACCACCATGAT |
| TLR6 | CACAGACAGCTTTGTACACCGTG | TGTGCTTGGTGCATGAGGA |
| TLR7 | CTCCATGCCATCAAGAAAGTTGA | GTTGAAGAGAGCAGAGCATGAG |
| TLR8 | GCTACAGGTCTCTTTCCACATC | GGTGGTAGCGCAGCTCATTTA |
| TLR9 | ACACAGCTGCGCAAGCTTAAC | TCGAGTGAGCGGAAGAAGATG |
| IRF3 | GCCCTTCATTGTAGATCTGATTACCT | CTTGACCATCACGAGCCTCTT |
| IRF7 | GCTCCCCACGCTATACCATCTAC | GCCAGGGTTCCAGCTTCAC |
| STAT1 | TGCATCATGGGCTTCATCAGC | GAAGTCAGGTTCGCCTCCGTTC |
| MxA | TTCAGCACCTGATGGCCTATC | GTACGTCTGGAGCATGAAGAACTG |
| IP-10 (CXCL10) | CGCTGTACCTGCATCAGCAT | CATCTCTTCTCACCCTTCTTTTTCA |
| VCAM-1 | CCGAAAGGCCCAGTTGAAG | AGCACGAGAAGCTCAGGAGAA |
| BAFF | TCTGGTGACTTTGTTTCGATGTATTC | GTTTTGCAATGCCAGCTGAA |
| Col IVa | GTGGATCGGCTACTCTTTTGTGAT | AATGGCGCACTTCTAAACTCCTC |
| IFNβ | ACAGACTTACAGGTTACCTCCGAAAC | CTCCTAGCCTGTCCCTCTGGGACTGG |
| 18S RNA | CCGAGTAAGTGCGGGTCATAA | CCATCCAATCGGTAGTAGCG |
